# Three Years with the Army of the Potomac—A Personal Military History

**Published:** 1912-01

**Authors:** William Warren Potter

**Affiliations:** Buffalo, N. Y., Brevet Lieutenant Colonel, U. S. Volunteers; Surgeon in Charge of First Division Field Hospital Second Army Corps; Surgeon 57th Regiment New York Volunteers; Assistant Surgeon 49th Regiment New York Volunteers; Recorder Second Division Hospital Sixth Army Corps, etc., etc.


### Three Years with the Army of the Potomac—A Personal Military History

By William Warren Potter, M.D., Buffalo, N. Y., Brevet Lieutenant Colonel, U. S. Volunteers; Surgeon in Charge of First Division Field Hospital Second Army Corps; Surgeon 57th Regiment New York Volunteers; Assistant Surgeon 49th Regiment New York Volunteers; Recorder Second Division Hospital Sixth Army Corps, etc., etc.

1863.

During my visit home my wife and I went to Michigan to visit my mother at Okemos. While there I was invited to a supper given by Zachariah Chandler to the Legislature and other guests, to the number of five hundred, at the Benton House in Lansing, upon the occasion of his re-election to the U. S. Senate. At the termination of my leave, or the time I had allotted to be absent, viz., January 19, 1863, I started back for the Army. I lost my baggage on my homeward trip somewhere between Albany and New York, so I returned by the same route spending a day in Albany in the search for it. I finally found it at the Chambers Street depot in New York, which was then the terminus of the New York Central R. R., and arrived in Washington on the morning of the 21st. I reported immediately to the Surgeon General, who found no fault because I had been away. He ordered me to report to Captain DeRussy at the War Department for muster, and this officer requested me to call the next morning when the papers would be ready. I did as requested, and on Thursday, January 22, was mustered out as Assistant Surgeon to date January 21, and mustered in as Surgeon to date January 22, 1863. My next step was to visit the Paymaster-General's office for payment as Assistant Surgeon, for November and December and to January 21, 1863. I was there informed that my case could not be reached in turn until Monday, the 26th, which would compel me to remain in Washington till Tuesday.

I met A. C. Winter, of Lansing, Michigan, Paymaster U. S. Navy, at the National Hotel Sunday evening, the 25th, in company with Mr. Leach, late Member of Congress from the Lansing district, and whom I had met a few days before at Chandler's big supper. During my stay in Washington I met Dr. Hall, who had been ordered up from the Army (No leaves were granted at this time) to see his son Theodore, who was sick in the hospital as he supposed. He found, however, on his arrival that the poor boy had died, and now the problem was to get permission to take the body home. Theodore had enlisted in the 154th N. Y. Volunteers without his father's knowledge or consent, and, of course, the family were very much heart broken. No leaves of absence were granted to officers at this period, which was just at the termination of Burnside's career when everything was in a state of confusion, and no one could predict what would take place next. I proposed to Dr. Hall that we interview Senator James R. Doolittle of Wisconsin, whom I had known before the war when we lived in Wyoming County, and see what he could do in the premises. We met the Senator in the hall of the National Hotel, and followed him to his room where I introduced the doctor, and stated our errand as briefly as possible. Without a word in reply, the Senator sat down and wrote a letter to the Secretary of War, stating the case in detail and closing with these words: "Now, my dear Secretary, even the sternest rules of war must permit a father to bury his dead son." I remember them as well as though it were yesterday. Armed with this letter we proceeded to the War Department, where it immediately brought us an audience with the Secrtary, who, without hesitation, ordered a ten days' leave of absence for the doctor, and also pay for November and December. Dr. Hall was so relieved that he thanked me over and again, the tears rolling down his cheeks in great drops as he bade me good-bye.

I left Washington Wednesday morning, January 28, for the Army of the Potomac, reaching Belle Plain Landing at five o'clock P. M. in the midst of a most disagreeable snow-storm. It was impossible for me to proceed that night to the camp of the 49th, whither I was bound, so I found quarters for the night at the Landing through the courtesy of an officer of the Q. M. Department. The next day, the 29th, I sent up for my horse, but by the time I reached camp it was nearly dark. My baggage was not brought up until near dark of the 30th, which occasioned another day's delay. It will be impossible for me to undertake a description of the roads at this time, but it is sufficient to state that the Army of the Potomac is literally, completely and totally stuck in the mud. It requires a whole day for teams to go to Belle Plain and return to White Oak Church with eight bags of grain, a distance of four miles and back, and yesterday they did not bring up any grain excepting on horseback.

A change of commanders has again taken place. Hooker relieves Burnside in command of the Army, Franklin goes away altogether, and Smith is also given another command. Colonel Bidwell is very kind to me, and Major Ellis is as gay and happy as ever. I enjoyed the two days spent with the 49th at this time very much indeed, and begin to dread the separation which must now be final.

I started for the 57th regiment Saturday morning, January 31, and reached there about noon, calling at Division Headquarters on the way where I saw Major John Hancock, A. A. General, and others of the staff whom I had known in the Sixth Corps before General Hancock was assigned to the Second. General Hancock was away on leave, and Colonel S. K. Zook, of the 57th, was in command of the Division. I learned from him the location of the camp of the 57th, and about noon I reported to Lieutenant Colonel Chapman for duty, he being in command of the regiment. The officers are a very fine body of young men, and all received me very cordially, though are frank to express their disappointment in the failure of Dr. H. C. Dean, the Assistant Surgeon, to receive the promotion. Dr. Dean is very kind to me, and altogether I cannot be otherwise than pleased with my reception. The regiment is small, having only about 170 men present for duty, and today, February 2, they are for the most part busily engaged in constructing log huts, so that in a few days they will be very comfortably situated. There are very few on the sick list as the camp is pleasantly located on a sandy knoll, so we are not troubled with the mud as much as many other portions of the Army are.

February 5. The entire field and staff occupy a hospital tent as sitting room and mess-room, behind which and communicating with it is a wall tent, that I occupy as my sleeping apartment. I like the officers very much indeed, and hope my first impressions will continue. Lieutenant Colonel Chapman is a young looking man with smooth face and dark hair and eyes, about 27 years old, and a splendid disciplinarian. It is snowing today quite hard, and such a storm in the North would soon make sleighing; but here it only means more mud.

February 8. This is my second Sunday with the 57th. Last Sunday Dr. Dean took me over to Corps headquarters, and introduced me to the Medical Director, Dr. Taylor, who is a brother of Bayard Taylor. Dr. Nelson Neeley, our 2d Assistant Surgeon, is a character—tall, phlegmatic, slow, but bright and dry with his wit. Yesterday we moved our camp about four hundred yards, and are very pleasantly situated. I have a nice old-fashioned fireplace in my quarters, that is now giving forth large dividends of both light and heat. I have a hospital tent for my front room, and a wall tent for my sleeping apartment, which is no longer used in common with the other officers as a mess-room. My large apartment is, however, a convenient rendezvous for the officers at evening, and they congregate there for social and friendly intercourse. Indeed, it is quite like a club room in the evening. Lieutenant Colonel Hammill, Dr. Wood, Dr. Vander Veer, Captain Derickson, and other officers of the 66th, as well as some from other regiments, frequently join us in our evening's entertainments, and we make up quite a merry party.

February 12. The times are very dull in camp these days, as the roads are so bad that riding is not agreeable, so we are committed to the dull routine of eating, reading, and sleeping in the daytime; but in the evening we have quite jolly times. Some of the officers are very good singers, among whom may be mentioned Captain Wright, Lieutenant Middleton, and Colonel Hammill of the 66th, who is always willing to lend his voice to aid in the entertainment of our visitors. Captain Rasdurichen, a Russian officer on General Hooker's staff, is a frequent visitor, and he occasionally passes the night with me, as I have an extra bed for guests.

February 15. I have been suffering with the toothache for a day or two. Today, however, the pain is gone, but my face is very much swollen; it is a reminder of the time I had at Harrison's Landing last summer. The officers are very kind to me, and treat me as though I was an acquaintance of many years, instead of a comparative stranger. Many officers are now obtaining leaves of absence for ten days, under an order permitting a certain number from each regiment to be away at a time. This is Monday, and on Friday last I visited the 49th, where I spent a part of the day very pleasantly. Calonel Bidwell had gone home for ten days, and Dr. Hall had not returned from the sad mission which called him home. When General Smith was ordered away from the Sixth Corps, the officers of his old Division, the 2d, paid him a visit in mass to bid him good-bye and God speed. He is very much liked by them all, and a movement is on foot to present him with a set of silver plate to cost $1,000.00. On Saturday last I visited Stafford C. H., where I saw the 136th, and 154th regiments. I there met Gad Parker, Daniel Post, and Levi Vincent; also Colonel Wood, who commands the 136th. At the 154th I called upon Dr. Van Aernam and Dr. Dwight W. Day. The weather has been more favorable for riding for two or three days past, and I have been improving the opportunity.

February 19. We have just passed through one of the most severe storms that I have experienced in Virginia. It snowed Monday night and all day Tuesday, and by Tuesday night we had over a foot of snow. It then set in and rained, the rain continuing all night Tuesday and through the day Wednesday and Wednesday night, ceasing about ten o'clock this (Thursday) morning, and now the mud is ankle deep or over. Dr. Hall came to see me Monday, the 16, and is feeling very much depressed from the loss of his son.

February 22. This being Washington's Birthday, salutes are being fired at several points within the lines—at General Hooker's Headquarters, Stafford C. H., Acquia Creek, and Belle Plain. It snowed all night last night and a good part of today, with high wind this P. M., and the snow is now drifting badly. It was so cold this morning that I did not get up until noon, as I could not get my fire started, and I found about six inches of snow in my tent when I awoke.

February 27. The roads are very bad and it is very dull in camp as a consequence. We cannot pay visits nor receive them, until we get some drying weather. Under the order granting leaves to a certain number of officers at a time, I may be able to go home next month for ten days. We shall see.

March 1. I visited the 49th yesterday and saw, among others, Colonel Alberger who was paying a visit to the old regiment. He is in the Veteran Reserve Corps now on account of his wound, which renders him unfit for field service. We were put through our rgular bi-monthly inspection and muster for pay yesterday, which fell on Sunday this time.

March 3. Today was appointed for a review by General Hooker, but it was postponed on account of the weather. I saw, and had a pleasant visit with Colonel Bidwell at the 49th the other day. He appears to be in much better health, and has resumed his usual urbanity of manner.

March 5. General Hooker reviewed the 2d Corps today. The troops looked well, though their ranks are thin. After the review we entertained a number of visiting officers in our (my) quarters.

March 10. The weather is disagreeable again, and a damp snow is falling. My horse is looking finely, as I have a good groom in the person of Patrick Corbleigh, one of the 57th soldiers and an old stable man from New York City.

March 13. Captain Rasdurichen, of General Hooker's staff, has been staying with me for two days. He is an agreeable companion and a friend of several officers of the regiment. Yesterday, the 12th, (Thursday), a wedding took place in the Third

Corps, which attracted great attention from the novelty of the affair. Captain de Hart, of the 7th New Jersey Volunteers, was married to Miss Lamond, of Washington, and it was made an occasion of great festivity in his regiment. A large platform was erected covered in on three sides with evergreen trees and boughs, upon which the ceremony took place, in the presence of General Hooker and staff, two other Major Generals, ten Brigadiers, and a large concourse of officers, soldiers, ladies, and civilians. Mollie Lewis, of Washington, whom I met several times at Mrs. Ridgeley's, was one of the bridesmaids. The band played "Hail to the Chief" on the arrival of General Hooker, and, amidst displays of bunting, salvos of artillery, and bursts of music, the two were made one. Leave of absence for twenty days was granted the groom, and the party goes to Washington by special boat after the festivities are over.

March 16. Tomorrow will be St. Patrick's Day, and great preparations are being made, for its celebration by the Irish Brigade which belongs to this Division, and whose camp adjoins ours. General Meagher is first and foremost in the affair, and we are expecting many visitors from other portions of the Army at our camp during the day.

March 18. Yesterday the celebration came off as expected and, it being a beautiful day, everything conspired to make it as jolly as possible. Horse races, the soaped pole, greased pig, and sack-racing were among the features of the day; and after these were concluded all were invited to General Meagher's headquarters to luncheon and arrack. The bridal party of last week was in attendance, and I had the honor of an introduction to the bride by Mollie Lewis. Today the party goes to Washington, and thence North. Major Ellis and Lieutenant-Colonel Theodore Hamilton, of the Sixth Corps, and other officers of that Corps, besides many who were friends of the 57th officers, were entertained in my quarters after the races, and the fun was kept up until a late hour of the evening.

March 20. It is cold and snowy today, and it is difficult to keep a comfortable fire on account of the changeable nature of the wind. There has been a cavalry fight somewhere on our right, as we heard distant firing at intervals during yesterday.

March 23. It is a year today since we sailed from Alexandria for the Peninsula. What changes have been wrought in that time! Who can tell what another year will bring forth? Today it is warm, with threatening rain, and the snow is all gone. Dr. H. B. Miller, of Alexander, New York, paid me a visit yesterday, which I enjoyed very much. He is spending a few days in the Army, and is quartering with the 136th New York, at Stafford C. H. On the 13th inst. I made application for leave of absence, but it was returned on the 14th disapproved. Today, as it was intimated that it would go through, I renewed it.

March 26. My application for leave came back today approved, authorizing me to be absent for ten days from the 27th, so I started on Friday morning, March 27, and reached Lancaster on Saturday night, the 28th, persuading the conductor to let me off, though the train did not make a regular stop there. At the expiration of my leave, not being able to return, I forwarded a Surgeon's certificate of disability to cover twenty days more; and, on the 20th of April, I started back, reaching Washington on the morning of the 21st. I went immediately to the Pay Department to obtain my pay to March 1, but Major Potter, who pays our Brigade, had gone down to the front for that purpose. On reaching the camp, April 22, I found I could not draw pay until a military commission had settled the question as to the cause of my absence, whether it was a good and sufficient one or not. This is in accordance with recent orders from the War Department in reference to officers absent on Surgeon's certificate of disability. When I reached Brook's Station, the nearest one to our camp, on my return I walked over, a distance of two miles and, as it was a very hot day, I was very much overcome on reaching my quarters. The regiment was in the same location, and nothing of importance had transpired during my absence, so I had lost nothing by my month's visit home. Now, however, everything looked like an immediate move, orders of a preparatory nature being constantly received. This is Wednesday, April 22, and I should not be surprised if we had a battle before the week ends. This is the impression that I reach from conversation with Colonel Chapman and other officers, whose judgment is reliable.

April 24. We are still in a state of disquiet, orders upon orders being received, but the final one to start has not been issued yet. While I was home we heard of the death of Captain McLean, Fifth U. S. Cavalry, which occurred suddenly in Washington, and rumor had it that he died in a debauch. This seems to have been the report made by Surgeon Basil Norris, U. S. Army, who was called, but did not see the Captain during life. This report prevented his widow from obtaining pension, and after the war I obtained a correction of the record upon representing to Dr. Norris the soldierly qualities of McLean, and his sabre wounds in the head, received during Stuart's raid around the Army of the Potomac, June 13, 1862. I procured a letter from General Custer substantiating my statements, whereupon Dr. Norris revoked his former report, and the pension was duly granted. This was during 1866 and 1867.

To return from this digression: I found the regiment in good health on my return and all were glad to see me back, finding no fault because I had prolonged my stay. Sunday, April 26, still on the qui vive, expecting to move at any moment; the weather is warm, and everything is in readiness for the start.

(*To be continued.*)

